# Light Emission from Single Oxygen Vacancies in Cu_2_O Films Probed with Scanning Tunneling Microscopy

**DOI:** 10.1021/acs.jpclett.3c00642

**Published:** 2023-04-21

**Authors:** Alexander Gloystein, Mina Soltanmohammadi, Niklas Nilius

**Affiliations:** † Carl-von-Ossietzky University, Institute of Physics, D-26111 Oldenburg, Germany

## Abstract

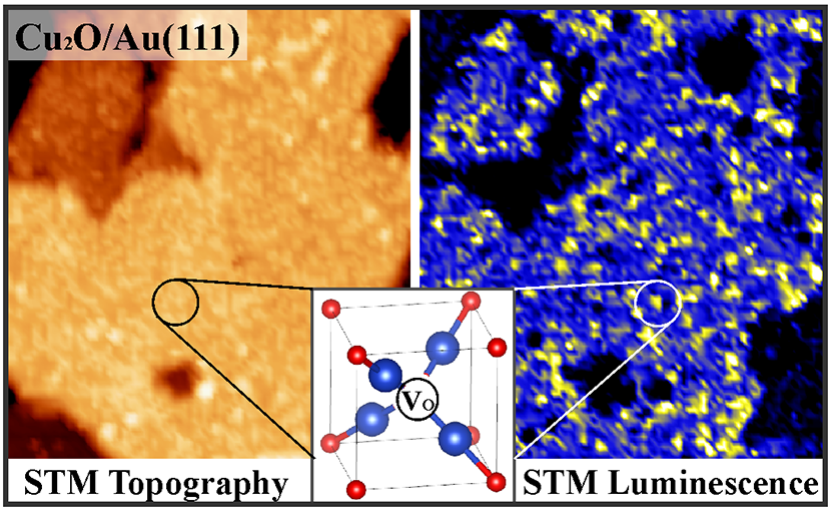

Global photoluminescence (PL) and spatially resolved scanning tunneling microscopy (STM) luminescence are compared for thick Cu_2_O films grown on Au(111). While the PL data reveal two peaks at 750 and 850 nm, assigned to radiative electron decays via localized gap states induced by O vacancies, a wide-band emission between 700 and 950 nm is observed in STM luminescence. The latter is compatible with cavity plasmons stimulated by inelastic electron tunneling and contains no spectral signature of the Cu_2_O defects. The STM luminescence is nonetheless controlled by O vacancies that provide inelastic excitation channels for the cavity plasmons. In fact, the emission yield sharply peaks at 2.2 V sample bias, when tip electrons are resonantly injected into O defect states and recombine with holes at the valence-band top via plasmon stimulation. The spatially confined emission centers detected in photon maps of the Cu_2_O films are therefore assigned to excitation channels mediated by single or few O vacancies in the oxide matrix.

The functionality of oxide materials is intrinsically connected to their defect landscape.^1^ This relevance arises from the high level of chemical saturation of ideal oxide lattices, as reflected in many secondary properties such as wide band gaps, low carrier densities, optical transparency, and chemical inertness. In fact, oxide defects often control the physical and chemical response of oxides, as they give rise to unsaturated chemical bonds, charge trapping, localized gap states and uncompensated spins. Defective oxides are therefore active in adsorption and catalysis; they develop p- or n-type conductance behavior and exhibit absorption and luminescence bands due to long-lived optical excitations. Even multiferroic properties can be implemented by tuning the defect landscape of oxides. A thorough microscopic and spectroscopic exploration of lattice defects is therefore paramount to rationalize the nature and functionality of oxides.

The classical approach to characterize oxide defects is optical spectroscopy. Already 100 years ago, researchers linked the intense luminescence from oxides that were exposed to electrons or X-rays to the formation of point defects in their lattice. Due to their unique emission response, they were termed color or F-centers, and tentatively assigned to neutral and charged oxygen vacancies.^2^ The atomic nature of these defects has been elucidated later by theoretical approaches^3^ and is nowadays well understood for most wide-gap oxides, e.g., MgO,^4,5^ Al_2_O_3_,^6^ CaO,^7^ SiO_2_^8^ and ZnO.^9^ Note that the concept of color centers also applies to impurities in the oxide lattice, i.e., cationic or anionic substitutions, that generate pronounced emission lines as well.^10,11^ More recently, electron paramagnetic resonance spectroscopy has evolved to an alternative tool to study defects and impurities in oxides.^12,13^

A main drawback of the above techniques to examine oxide defects is their limited spatial resolution that conflicts with the large impact of the direct atomic environment on defect properties. Given the poor screening in dielectrics, the symmetry of adjacent atoms, the local charge distribution and the dynamic relaxation behavior of the surrounding lattice drastically affect the electronic and optical response of oxide defects. Unfortunately, possibilities to probe individual defects in their true atomic neighborhood are limited. Scanning probe techniques, especially STM and AFM, play a central role in detecting structural perturbations around lattice defects, as impressively demonstrated for O vacancies in NiO,^14^ TiO_2_,^15^ CeO_2_,^16,17^ and MgO surfaces.^18^ In some cases, the structural data could be complemented with electronic information, proving the correlation between defects and confined gap states. Spectroscopy of local defect states was reported for point and line defects in MgO,^18,19^ Al_2_O_3_,^20^ and ceria,^21^ among others. Finding an experimental link between the structural and optical response of individual defects is even more challenging. Promising results in this regard were achieved by cathodo-luminescence spectroscopy, using the confined electron beams of electron or tunneling microscopes as a source of excitation. By this means, the optical response of small ensembles of color centers (MgO, ZnO^22^) and impurity ions (Al/ZnO,^23^ Fe/Al_2_O_3_^24^ and Cr/MgO^11^) has been probed together with their local atomic structure. Although optical characterization of single oxide defects has not been achieved so far, promising results were reported for other dielectrics, e.g., N-vacancies and color centers in diamond^25^ and hBN,^26^ respectively.

In this work, we present STM luminescence data for oxygen vacancies (V_O_) in cuprous oxide (Cu_2_O) films grown on Au(111). Hereby, emission centers of ∼1 nm diameter are detected that are compatible with individual color centers. As the defects locate in subsurface positions, they are not directly resolved in the STM images. A unique bias dependence of the emitted intensity marks, however, the position of the V_O_ defect states in the Cu_2_O band gap.

Figure 1 presents two STM images of the Cu_2_O(111) film, being characterized by grains of 25–50 nm size and ∼2 nm height. The film morphology is largely controlled by the annealing conditions, with higher temperatures resulting in better shaped crystallites yet higher defect densities. The oxide top facets are homogeneously covered with protrusions of ∼1.5 Å height and ∼10.5 Å periodicity. Each maximum marks a Cu_4_O unit of the nanopyramidal reconstruction, being the thermodynamically preferred termination of Cu_2_O(111) and responsible for its (√3 × √3)R30° superstructure.^27^ In the reconstruction, every third Cu–O six-ring of the bulk-cut surface gets occupied by a Cu_4_O unit that chemically saturates all oxygen dangling bonds and leads to a substantial decrease of the surface energy. At the employed preparation conditions, the reconstruction shows only moderate long-range order, while more regular arrays of Cu_4_O pyramids can be prepared at higher temperature, yet with undesired effects on defect density and luminescence response.

**Figure 1:**
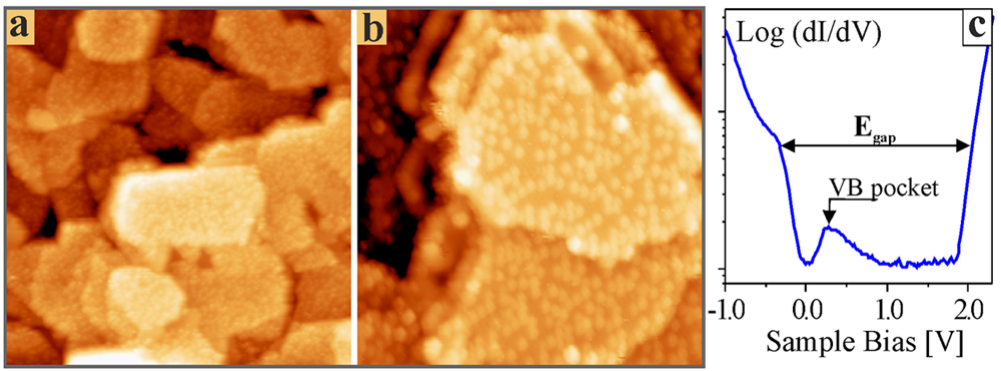
(a) Overview (100 × 100 nm^2^, *U*_B_ = 3.0 V, *I* = 0.1 nA) and (b) high-resolution STM topographic image (40 × 40 nm^2^, *U*_B_ = 2.0 V) of Cu_2_O/Au(111) films. (c) Constant-height d*I*/d*V* spectrum taken at 2.5 V and 0.1 nA set point current.

Figure 1c displays a d*I*/d*V* spectrum of the Cu_2_O film, shown in logarithmic representation to highlight small conductance features. The dominating feature is the oxide band gap, extending from the valence-band (VB) top at −0.1 V to the conduction-band (CB) bottom at +1.9 V. The gap width of 2.0 eV agrees well with reported values for bulk Cu_2_O.^28^ The VB top is pinned to the Fermi level, in-line with a robust p-type conductance behavior of the oxide.^29^ The in-gap peak at 0.3 V arises from an empty-state pocket at the VB top, induced by an upward bending of the oxide bands in response to the Cu-deficient nature of the nanopyramidal reconstruction.^30^ The kink at −0.3 V reflects the flat-band situation in the surface that is reached at negative sample bias.

The global PL signature of the Cu_2_O films upon illumination with 532 nm photons is depicted in Figure 2a. The spectrum reveals an asymmetric band that can be decomposed into two Gaussians centered at 750 and 850 nm. According to earlier work, they are assigned to the emission from double- (V_O2+_) and single-charged (V_O+_) oxygen vacancies in the lattice.^31,32^ Their intensity ratio depends on the hole concentration in the oxide VB, whereby the strong p-type character of Cu_2_O/Au(111) promotes formation of V_O2+_ defects and leads to a more intense 750 nm peak.^33^ Other PL peaks intrinsic to bulk Cu_2_O, especially the free-exciton emission at 620 nm and the V_Cu_ mediated peak at 920 nm, are not detected here because of the finite crystallinity, i.e., the abundance of nonradiative decay channels, in the supported films.^34^ In fact, long excitation lifetimes are required to detect the dipole-forbidden Cu_2_O excitons, rendering them invisible in typical nanocrystalline films and powders.^35^ The same applies to the V_Cu_ luminescence that gets initiated by exciton trapping at Cu defects. In contrast, the V_O_ emission relies on hot electrons in the oxide CB and features much shorter radiative decay times than the exciton-mediated channels. Consequently, it is less sensitive to structural disorder and governs the PL response of Cu_2_O samples of low crystallinity.

**Figure 2:**
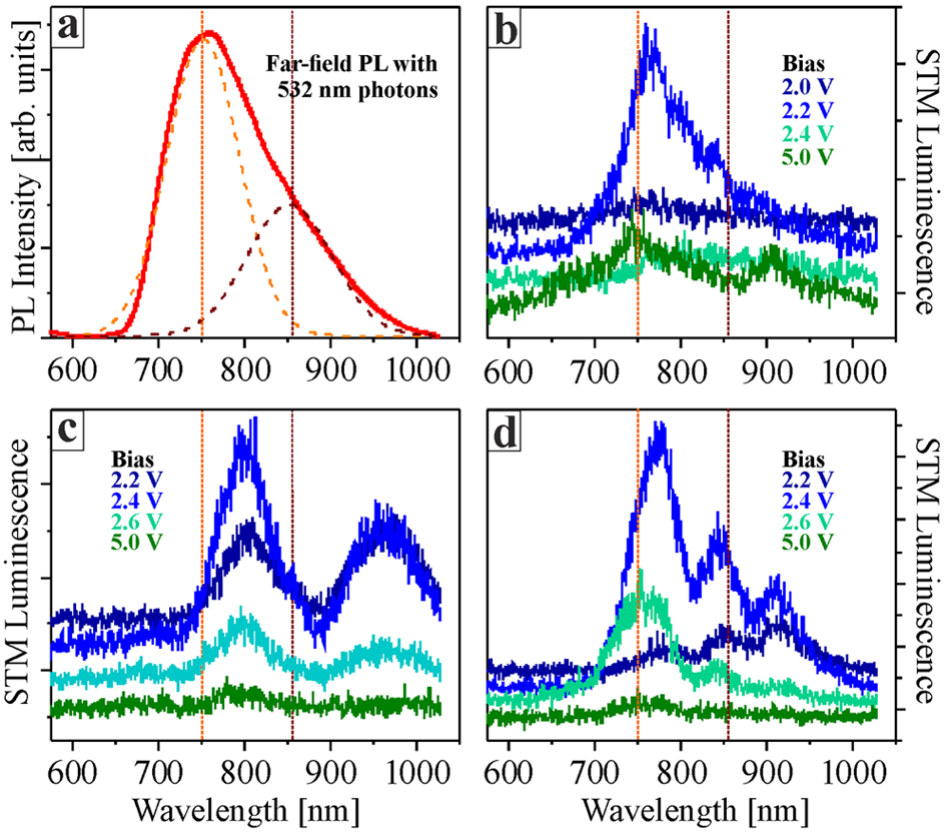
(a) PL spectrum of a 10 nm Cu_2_O/Au(111) film (100 K, 60 s accumulation time). (b–d) STM luminescence spectra of three different films taken at the indicated bias values (100 K, I = 1 nA, 180 s accumulation time, offset for clarity).

The local emission response, stimulated by electron injection from the STM tip into well-shaped Cu_2_O grains, is depicted in Figure 2(b–d). The spectra were acquired with different tip configurations on three oxide films, yet at similar excitations conditions (1 nA current, 2.0–5.0 V sample bias). All series follow the same spectral envelop, characterized by an emission onset at 700 nm, a first peak below 800 nm and a nonmonotonous decay at higher wavelength. Details of the spectra vary distinctively, however, and one, two and three emission maxima are discernible in the series shown in the panels (b) to (d). Surprisingly, the spectral signature within one run is weakly bias-dependent and remains constant even at negative polarity, i.e., for electron tunneling from the sample to the tip. Conversely, the emission intensity changes distinctively with bias voltage. It runs through a sharp and universal maximum at +2.2 V and declines with lower and higher bias. In all cases, the emission maxima of the far-field PL, marked by dashed lines in Figure 2, are not reproduced in the near-field spectra.

To gain quantitative insights into the bias-dependence of the STM luminescence, the integral photon intensity was probed with a photomultiplier (Figure 3). For this purpose, the sample bias was ramped from 4 to 1.5 V with enabled feedback loop (*I* = 1 nA) and luminescence, tip-height and d*I*/d*V* signals were simultaneously recorded. The pronounced emission maximum at 2.2 V is readily seen in the blue curve. It locates just below the CB onset at 2.3 V, the latter being identified by a rising tip height and d*I*/d*V* signal as new electronic states become available for tunneling. Note that the CB onset detected in Figure 3 is higher than in the d*I*/d*V* curve of Figure 1c, which is explained with a stronger tip-induced band bending in the luminescence experiments performed at a 10× larger tunneling current. Once the CB onset is reached, the emitted intensity drops sharply from 2000 to 100 counts/s (Figure 3). Such a decline seems incompatible with diminishing field-enhancement effects in the STM cavity, as induced by the gradual tip retraction from the surface at higher bias. It rather suggests resonant tunneling into a localized light emitting state inside the oxide band gap.

**Figure 3:**
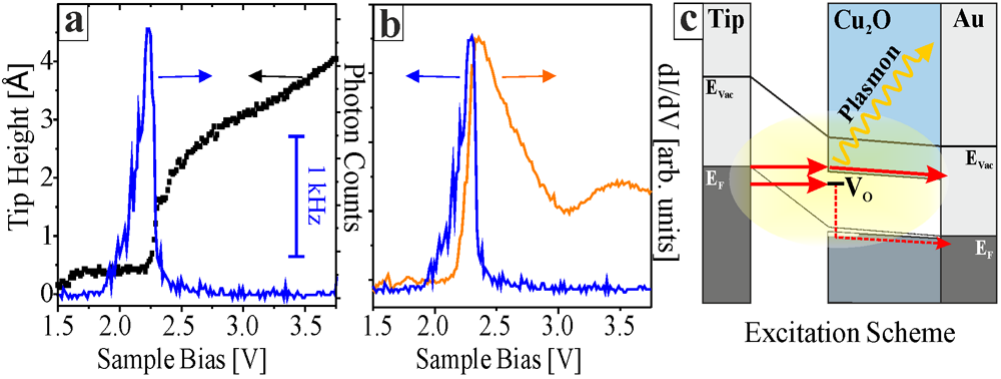
(a) Integral emission yield probed with a photomultiplier and simultaneously measured tip-height during a constant-current ramp from 4.0 to 1.5 V (*I* = 1 nA). (b) Same emission data as in (a) but shown together with the d*I*/d*V* signal. (c) Potential diagram of the STM junction containing a Cu_2_O/Au(111) film. Solid and dashed arrows depict the elastic and inelastic electron transport channels, respectively. The latter initiates plasmon-mediated light emission from the STM cavity.

To complement the experimental section, luminescence maps of the Cu_2_O surface were acquired with the photomultiplier (Figure 4). While the map in (b) was recorded at the emission maximum at 2.2 V, the one in (c) represents an off-peak bias and shows reduced intensity. Two conclusions can be drawn from these photon maps. (i) Light emission at 2.2 V is only detected from thick Cu_2_O grains, and holes in the film, exposing a Cu_3_O_2_ monolayer,^36^ are optically inactive. And (ii), the Cu_2_O luminescence is very heterogeneous in space, with bright centers alternating with dark regions. The effect becomes even clearer in high-resolution maps (Figure 4, lower panels). Apparently, the emission originates from localized spots of ∼1 nm size, being randomly distributed in the oxide surface (Figure 4e). While most emission spots are spherical, some exhibit complex shapes due to agglomeration of adjacent optical centers. The simultaneously recorded topographic and d*I*/d*V* maps are displayed in panels (d) and (f). In a search for common patterns, characteristic luminescence (triangles, right half) and electronic features (circles, left half) are marked in all three channels of the measurement. However, the triangular and spherical symbols seem uncorrelated and topographic peculiarities in the surface do not coincide with regions of pronounced optical or electronic activity. With other words, the inelastic (luminescence) and elastic (conductance) tunneling channels do not probe identical regions in the Cu_2_O surface.

**Figure 4:**
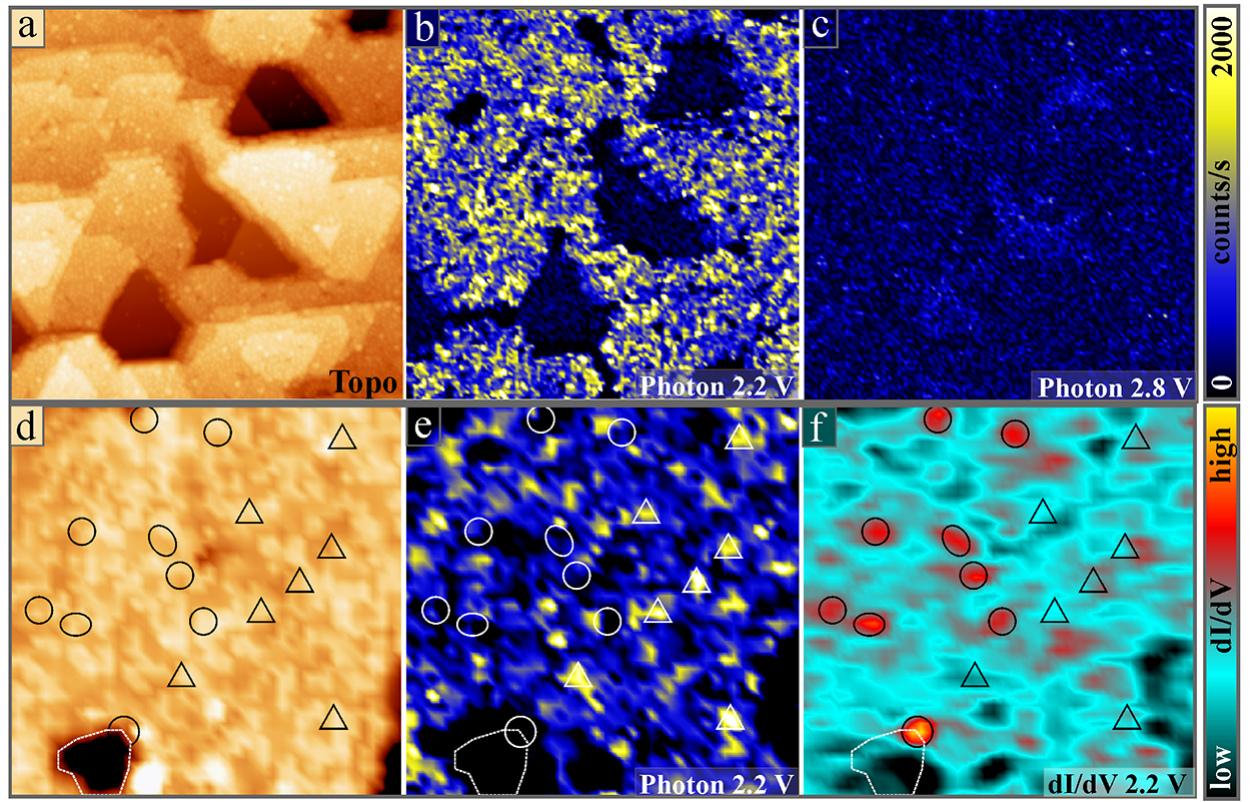
(a) Topography and associated photon maps of a Cu_2_O/Au(111) film taken at (b) 2.2 V and (c) 2.8 V sample bias (100 × 100 nm^2^, *I* = 1 nA). Better resolved data for a similar film, (d) topography, (e) photon map and (f) corresponding d*I*/d*V* map (25 × 25 nm^2^, *U*_B_ = 2.2 V, *I* = 1 nA). The triangles and ellipses mark characteristic emission and electronic features, respectively.

The following paragraphs will discuss why far-field PL and local STM luminescence spectra of our Cu_2_O/Au(111) films appear different. Although not obvious at first glance, we will show that V_O_ color centers are responsible for the emission response in both cases and that light emission from single oxygen vacancies can actually be detected in our experiments.

The PL signature of V_O2+_ and V_O+_ complexes in Cu_2_O are peaks at 750 and 850 nm, broadened to 80–100 nm FWHM due to finite sample crystallinity.^33,34,36,37^ The underlying decay channels were assigned by density functional theory (DFT) to in-gap resonances at 1.6 and 1.5 eV above the VB top, introduced by V_O2+_ and V_O+_ defects, respectively.^38^ While V_O2+_ centers preferentially develop in highly p-type oxides with the VB top pinned to *E*_F_, as in our case, any downshift of the VB promotes the formation of single-charged V_O+_ defects. Our detected STM luminescence deviates markedly from this V_O_ emission response, as it covers a much wider wavelength range from 700 to 950 nm and depends sensitively on the actual tip state. However, this emission signature follows the one of cavity plasmons, to be viewed as collective and coherent oscillations of the electron gases in tip and sample stimulated by inelastic electron tunneling in the STM.^39,40^ In our case, the spectral response of the plasmons is governed by the dielectric function of the Au tip and the Au substrate,^41^ while the weakly polarizable oxide layer leads only to a minor red shift of the plasmon mode. The tip apex, on the other hand, plays a decisive role and its microscopic polarizability defines the fine structure of the plasmon emission. This unique tip dependence is readily deduced from the emission series shown for three equivalent Cu_2_O/Au(111) films in Figure 2. Moreover, the spectral behavior within one series is rather insensitive to bias voltage and polarity, fulfilling other criteria of cavity plasmons.^40,41^ Apparently, the optical characteristics of the V_O_ defects is not reflected in the STM data, which might be explained with the low oscillator strength of single-electron defect transitions with respect to collective plasmon excitations. Even though the V_O_ defects leave no fingerprint in the spectral response, they play a central role for exciting the plasmons in the STM cavity.

As presented in Figure 3, the luminescence yield runs through a pronounced bias maximum at 2.2 V that is below the actual CB onset. At this condition, elastic tunneling between tip and sample is largely suppressed, as electrons have not enough energy to reach the oxide CB, while empty states in the Au(111) support are too far away. An inelastic tunneling pathway seems, widely open, however, producing the observed emission peak. We assign this path to resonant tunneling into V_O_ defect states in the Cu_2_O band gap, followed by an inelastic decay to the empty state pocket at the VB top (Figure 3c). The energy released in this process is transferred to STM cavity plasmons and triggers the detected luminescence signal. Support for this interpretation comes from the bias position of the luminescence maximum that needs to be corrected, however, for band-bending effects in the tip electric field. By employing a correction scheme that shifts the CB onset measured at 1 nA (2.3 V, Figure 3) to its low current position (1.9 V, Figure 1), a defect position of 1.7 V is obtained, in good agreement with the calculated V_O2+_ resonance energy.^39^ Another argument for a defect-induced inelastic transport channel is the sharpness of the intensity maximum on the bias scale. While below 2.0 V, no V_O_ gap states can be reached, although defects in different oxide layers may have different energy, the opening of elastic channels above 2.3 V abruptly quenches the inelastic tunneling path. The detection of V_O_ mediated light in our study thus results from a fortunate interplay of effective inelastic tunneling via plasmon stimulation in absence of elastic transport paths.

The size of individual emission spots in our photon maps can be as small as 1 nm, only slightly larger than the theoretical extension of V_O_ defect states that cover the actual vacancy and four adjacent Cu ions.^39^ We therefore conclude that light emission from single O vacancies can be detected. The distribution of defects in the Cu_2_O films seems random and does not follow a distinct pattern. Moreover, emission centers in the photon maps do not leave fingerprints in the STM topography, as visualized by the triangles in Figure 4d. The O vacancies responsible for inelastic tunneling might thus be located in subsurface regions of the oxide, in agreement with the thermodynamic instability of O defects in the Cu_2_O(111) surface found by DFT.^28^ Also, the anticorrelation of optical centers in the photon maps and sites with high d*I*/d*V* intensity is remarkable (compare triangles and circles in Figure 4e,f). It emphasizes once more that pronounced optical activity is only detected when competing elastic tunneling channels are suppressed. The spots of high d*I*/d*V* signal are tentatively attributed to oxide regions with downshifted CB onset, for instance due to smaller surface band bending. In these regions, elastic tunneling becomes activated already at lower bias and inelastic channels are less relevant.

In summary, while the far-field PL of Cu_2_O/Au(111) films is governed by two distinct peaks at 750 and 850 nm, related to electron recombination via V_O2+_ and V_O+_ defect states, multiple peaks between 700 and 950 nm are detected in STM luminescence. The latter follows a near-field emission scheme mediated by cavity plasmons, in which Cu_2_O defects develop no spectral fingerprint. The V_O_ defects still play a central role for plasmon excitation in the STM, as they provide inelastic tunneling channels through the Cu_2_O band gap where elastic tunneling is inefficient. A sharp maximum in the luminescence yield at 2.2 V marks the energy position of the V_O_ gap states. Moreover, photon maps taken at this bias reveal the spatial distribution of individual oxygen vacancies in the Cu_2_O film. To access the V_O_ luminescence also in the spectral regime, the impact of cavity plasmons needs to be diminished, for example, by using nonplasmonic substrates and tip materials. Whether inelastic tunneling remains effective enough to detect the local defect emission also in this case needs to be explored in upcoming experiments.

## Experimental Methods

The experiments have been performed in an ultrahigh vacuum chamber equipped with a Beetle-type STM operated at 100 K and standard tools for sample preparation and thin-film deposition. Photon emission from the tip–sample junction was collected with a lens system and detected either with a Peltier-cooled photomultiplier or a charge-coupled device for integral and spectral analysis, respectively. Differential conductance (d*I*/d*V*) spectra were acquired with a lock-in amplifier at ∼14 mV modulation bias and ∼1.8 kHz frequency. An electrochemically etched gold wire was used as the tip. Conventional photoluminescence (PL) data were recorded with the 532 nm photons of a Nd:YAG laser at 100 K.

The Cu_2_O films were prepared in a three step procedure:^27^ (i) Deposition of 10 nm Cu metal on a sputtered and annealed Au(111) single crystal. (ii) Oxidation in a designated high-pressure cell at 50 mbar O_2_ and 450 K. (iii) Crystallization at 600 K in 10^–4^ mbar O_2_ in front of a pinhole doser to avoid oxide reduction. The resulting films showed good crystallinity, as deduced from sharp, hexagonal (1 × 1) and faint (√3×√3)R30° superstructure spots in electron diffraction, the latter being in-line with the nanopyramidal reconstruction of Cu_2_O(111) (Supporting Information).^27^ The oxide stoichiometry was determined with X-ray photoelectron spectroscopy to be Cu_1.85_O; i.e., the films were slightly Cu deficient, in agreement with their p-type conductance behavior (Supporting Information).

## Supporting Information Available

Low-energy electron diffraction (LEED) and X-ray photoelectron spectroscopy (XPS) data of Cu_2_O films (PDF)
Transparent Peer Review report available (PDF)
