# Supplementary material for: Light Emission from Single Oxygen Vacancies in Cu2O Films Probed with Scanning Tunneling Microscopy
Source: J Phys Chem Lett. 2023 Apr 21;14(17):3980–5. doi: 10.1021/acs.jpclett.3c00642 (PMC10165647; doi:10.1021/acs.jpclett.3c00642)
Supplement: Supplementary file 1 — jz3c00642_si_001.pdf [file jz3c00642_si_001.pdf]

Supporting Information for

Light Emission from Single Oxygen Vacancies in Cu_2O Films Probed with the STM

Alexander Gloystein,[a] Mina Soltanmohammadi,[a] Niklas Nilius [a,*]
[a] Carl-von-Ossietzky University, Institute of Physics, D-26111 Oldenburg, Germany
*Email: niklas.nilius@uni-oldenburg.de, phone +49-441-798-3152

Figure S1 displays a Low-Energy-Electron-Diffraction (LEED) measurement of the 5 nm thick $Cu_2O(111)$ film grown on Au(111). The main (1×1) spots reflect the hexagonal symmetry and the 6.1 Å lateral periodicity of the oxide surface, both in agreement with respective literature data for bulk crystals.[1] The (√3×√3)R30° superstructure, on the other hand, is compatible with the nano-pyramidal reconstruction that was recently identified as energetically preferred surface termination of $Cu_2O(111)$.[2] In this structure, every third Cu-O six ring of the regular (111) surface is occupied by a Cu_4O nano-pyramid, saturating all oxygen dangling bonds in the top plane. As a result, the surface energy is substantially reduced, rendering the reconstructed surface thermodynamically preferred over the stoichiometric and any other surface model proposed for $Cu_2O(111)$ so far.

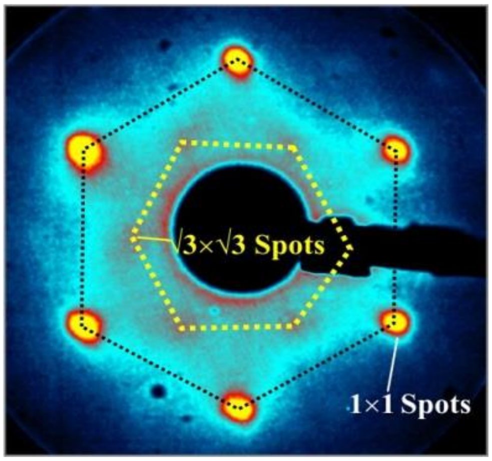

Figure S1: LEED pattern of a 5 nm thick Cu_2O film grown on Au(111) taken at E_{kin} = 37 eV.

To obtain insights into stoichiometry and composition of the Cu_2O/Au(/111) films, XPS measurements have been performed with an Al Kα source and the electron detector oriented in normal direction to the sample. Figure S2 depicts respective XP spectra taken in the Cu 2p, Cu L_3VV Auger and O 1s energy region, respectively. The detected spectral fingerprints are fully compatible with the ones of bulk Cu_2O.[3,4,5] In particular, no satellites are revealed in between the Cu $2p^{1/2}$ and $2p^{3/2}$ peaks that would be indicative for Cu^{2+} ions and CuO formation. Moreover, the presence of metallic Cu can be excluded from the single-peak structure of the Cu Auger line, as side bands at 918 and 920 eV kinetic energy should appear otherwise. Finally, only a single O maximum at 530.6 eV is detected, in agreement with the O 1s peak position reported for fully oxidized Cu_2O. A quantitative analysis of the XPS peaks, performed after Shirley background subtraction and correction for atomic sensitivity factors, reveals a Cu to O ratio of 1.85 for the Au(111)-supported films. The films are therefore Cu-deficient, in agreement with their pronounced p-type conductance behavior.

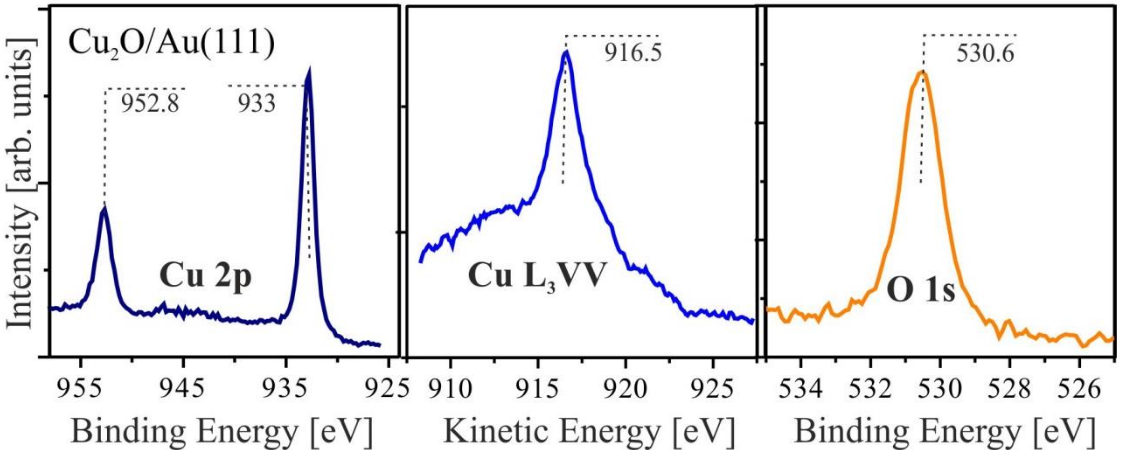

Figure S2: XP spectra of 5 nm thick Cu_2O/Au(111) films, measured in the energy range of the Cu 2p (left), Cu L_3VV Auger (middle) and O 1s peak (right).

---

[1] Meyer, B. K.; Polity, A.; Reppin, D.; Becker, M.; Klar, P. J.; Sander, T.; Reindl, C.; Benz, J.; Eickhoff, M.; et al. Binary Copper Oxide Semiconductors: From Materials towards Devices. *Phys. Stat. Sol.* **2012**, *249*, 1487-1509.

[2] Gloystein, A.; Nilius, N.; Goniakowski, J.; Noguera, C. Nanopyramidal Reconstruction of Cu_2O (111): A Long Standing Surface Puzzle Solved by STM and DFT. *J. Phys. Chem. C* **2020**, *124*, 26937–26943.

[3] Poulston, S.; Parlett, P. M.; Stone, P.; Bowker, M. Surface Oxidation and Reduction of CuO and Cu_2O Studied with XPS and XAES. *Surf. Interface Anal.* **1996**, *24*, 811–820.

[4] Tahir, D.; Tougaard, S. Electronic and Optical Properties of Cu, CuO and Cu2O Studied by Electron Spectroscopy. *J. Phys. Condens. Matter* **2012**, *24*, 175002.

[5] Maack, B.; Nilius, N. In-Situ Optical View onto Copper Oxidation – Role of Reactive Interfaces and Self-Heating. *Corros. Sci.* **2019**, *159*, 108112.
