# Supplementary material for: Light Emission from Single Oxygen Vacancies in Cu2O Films Probed with Scanning Tunneling Microscopy
Source: J Phys Chem Lett. 2023 Apr 21;14(17):3980–5. doi: 10.1021/acs.jpclett.3c00642 (PMC10165647; doi:10.1021/acs.jpclett.3c00642)
Supplement: Supplementary file 2 — jz3c00642_si_002.pdf [file jz3c00642_si_002.pdf]

jz-2023-00642d.R1

Name: Peer Review Information for "Light Emission from Single Oxygen Vacancies in Cu_2O Films Probed with the STM"

First Round of Reviewer Comments

Reviewer: 1

Comments to the Author

the author presented a manscript on the luminescence of O vacancy in Cu_2O film by STM technique, which is really interesting, in which they have assigned the emission band from the plasmon in this film. but there are still some evidences to be attached in the manuscript or Support information, for example, 1) the absorption spectra, especially in the range of near infrared 700-1000nm. 2) the XRD pattern of the film for the structure. 3) the XPS results on the Cu 2P to check the valence state and atomic ratio. moreover, the vacancy critical concentration for the local plasmon may be needed for its formation, and how the plasmon form and energy range ? what is the structural character for the individual state indicated in this manuscript?

hence the major revision is needed for its publication.

Author's Response to Peer Review Comments:

Response to Reviewer: 1

We are grateful to the reviewer for taking the time to read and evaluate our paper. We are very glad to see that the overall assessment of our work is positive. Nonetheless, we have carefully addressed all concerns addressed in the report and have improved our work where possible. Please find attached a point-by-point list of all criticized points:

(i) Reviewers comment: Attach to the supping information the absorption spectra, especially in the range of near infrared 700-1000nm.

(i) Authors response: We are unable to provide an absorption spectrum at this point, because our 5-nm thick film is grown on an opaque Au(111) crystal. We have tried reflectance spectroscopy as work-around, however, also this data is largely governed by the reflectance of the metal support. Non-local optical information is still provided by the luminescence spectra shown in Fig. 2a. The dominating response here is an emission band related to oxygen vacancies. Detailed insight into the non-local optical behavior of thin-film versus bulk cuprous oxide is provided in our earlier work (https://doi.org/10.1002/pssa.202200887).

(ii) Provide a XRD pattern of the film for the structure.

(ii) Authors response: Although we cannot provide XRD data of our films, as we lack the required UHV-compatible infrastructure, we have added electron diffraction (LEED) measurements in the supporting information (SI). This method is even more suited to generate structural insights, as it is extremely surface sensitive and probes the long-range atomic order of our film. The LEED pattern is composed of sharp (1 x 1) reflexes, reflecting the hexagonal surface unit cell of $Cu_2O(111)$ with 6.1 Ang periodicity, as well as (sqr3 x sqr3) superstructure spots. The latter arise from the nano-pyramidal reconstruction of the oxide film, in which Cu_4O nano-pyramids arrange with 10.5 Ang periodicity to passivate all dangling bonds in the surface. The superstructure reflexes are relatively broad, as not all nano-pyramids sit exactly at their correct position. However, higher annealing to improve structural order of the films was avoided here, as it strongly interferes with the defect landscape of the oxide.

Changes in the text: Adding electron diffraction pattern to the SI (Fig. S1).

(iii) Reviewers comment: Show XPS results on the Cu 2P to check the valence state and atomic ratio.

(iii) Authors response: We have included detailed XPS data of our Cu_2O/Au(111) film in the supporting information. The spectra cover the Cu 2p, O 1s and Cu LVV Auger peaks and thus allow for detailed insights into the oxide stoichiometry. The following information can be derived:

► Cu appears only in the 1+ oxidation state, as expected for Cu_2O. No Cu^{2+} species (identified by a pronounced satellite between the Cu 2p 1/2 and Cu 2p 3/2 peaks) and metallic Cu^0 components (identified by high-energy bands in the Auger spectra) are seen.

► By correcting the Cu 2p and O 1s peaks for their atomic sensitivity factors and dividing the respective peak areas, the stoichiometry of Cu2O has been determined to 1.85. Apparently, the film is Cu-deficient, in agreement with its p-type conductance behavior. However, it also contains O vacancies as evidenced by

the respective luminescence bands in the far-field PL. As Cu and O vacancies coexist in the oxide due to incomplete vacancy annihilation, the absolute defect concentration cannot be determined.

Changes in the text: Adding XP spectra of the film to the SI (Fig. S2).

(iv) Reviewers comment: Give the vacancy critical concentration needed for the local plasmon formation, and how the plasmon form and energy range?

(iv) Authors response: There might be a slight misunderstanding behind this question. The plasmon discussed in our paper is actually not an oxide plasmon due to free carriers in the Cu_2O film. Instead, the light emitting mode results from a cavity plasmon coherently excited in the free-electron gases of STM tip and Au(111) substrate below the film. This plasmon is excited by inelastically tunneling electrons and couples efficiently to light due to its large vertical dipole moment. The oxide film in the junction hereby acts as polarizable medium and induces only a small redshift to the plasmon frequency without imprinting its own spectral signature. Also, the low-density of O defects in our films has no consequence on the spectral response of the cavity plasmon.

Changes in the text: The role of STM cavity plasmon on the detected optical response is discussed in detail on p.11.

(v) Reviewers comment: What is the structural character for the individual state indicated in this manuscript?

(v) Authors response: For the structural and electronic nature of the O vacancy level in the band gap, we can only refer to respective DFT calculations (see Ref. 38). Here, a tetrahedrally coordinated O atom is removed from the lattice, causing the four direct Cu neighbors to relax slightly away from each other. The two electrons left behind by a desorbing neutral O atom may now annihilate with two holes that are abundant in the p-type oxide, producing a V(O2+) vacancy. Alternatively, only one electron may annihilate while the other one equally distributes among the four Cu nearest neighbors, resulting in a V(O+) defect. In both cases, the resulting defect state delocalizes over these four Cu cations surrounding the vacancy, yet with slightly different energies with respect to the valence band.

Changes in the text: The quantum mechanical nature of the V(O) defects is explained on the basis of DFT calculations on p.10.
